# Investigating the Strain, Temperature and Humidity Sensitivity of a Multimode Graded-Index Perfluorinated Polymer Optical Fiber with Bragg Grating

**DOI:** 10.3390/s18051436

**Published:** 2018-05-05

**Authors:** Yulong Zheng, Kort Bremer, Bernhard Roth

**Affiliations:** Hannover Centre for Optical Technologies (HOT), Leibniz University Hannover, 30167 Hannover, Germany; Kort.Bremer@hot.uni-hannover.de (K.B.); Bernhard.Roth@hot.uni-hannover.de (B.R.)

**Keywords:** perfluorinated polymer optical fiber, Fiber Bragg gratings, temperature, strain, humidity sensing

## Abstract

In this work we investigate the strain, temperature and humidity sensitivity of a Fiber Bragg Grating (FBG) inscribed in a near infrared low-loss multimode perfluorinated polymer optical fiber based on cyclic transparent optical polymer (CYTOP). For this purpose, FBGs were inscribed into the multimode CYTOP fiber with a core diameter of 50 µm by using a krypton fluoride (KrF) excimer laser and the phase mask method. The evolution of the reflection spectrum of the FBG detected with a multimode interrogation technique revealed a single reflection peak with a full width at half maximum (FHWM) bandwidth of about 9 nm. Furthermore, the spectral envelope of the single FBG reflection peak can be optimized depending on the KrF excimer laser irradiation time. A linear shift of the Bragg wavelength due to applied strain, temperature and humidity was measured. Furthermore, depending on irradiation time of the KrF excimer laser, both the failure strain and strain sensitivity of the multimode fiber with FBG can be controlled. The inherent low light attenuation in the near infrared wavelength range (telecommunication window) of the multimode CYTOP fiber and the single FBG reflection peak when applying the multimode interrogation set-up will allow for new applications in the area of telecommunication and optical sensing.

## 1. Introduction

Polymer optical fibers (POFs) comprising Fiber Bragg gratings (FBGs) are emerging as new fiber sensors that have potential to be used for the detection of temperature [1], strain [2], humidity [3] or other physical quantities [4,5]. In comparison to silica optical fibers, POFs display several advantages such as higher failure strain [6], flexibility in bending [7], and lower Young’s modulus [8], which provides enhanced sensitivity to strain and force, for example. Depending on the application, various types of POFs with specific characteristics can be fabricated by simply using different polymer materials with suitable doping. In terms of sensing applications, to date, the most investigated and widely used POF is based on polymethyl-methacrylate (PMMA) due to its high absorption sensitivity to deep-UV light and its low cost. The sensitivity to deep-UV light allows the inscription of FBGs into PMMA-based POFs by using, e.g., a KrF excimer laser and the established phase mask technique [9]. However, due to the vibrational absorption of carbon-hydrogen (C−H) bonds, PMMA has high optical losses in the near infrared region [10]. This feature makes PMMA unsuitable for long-distance telecommunication applications and POF-based sensing networks in the near infrared wavelength range. This drawback can be minimized by substituting the hydrogen of the carbon-hydrogen (C−H) bonds by heavier atoms [11]. A typical candidate is perfluorinated polymer, in which the carbon-hydrogen bonds are replaced with carbon-fluorine (C−F) bonds to shift the high absorption in the telecommunication windows at around 1300 nm and 1500 nm to the longer wavelength range and consequently reduce the inherent fiber attenuation in the telecommunication windows [12]. In recent times, one of the intensively investigated perfluorinated polymers is the cyclic transparent optical polymer (CYTOP) [13,14,15]. Today, multimode graded-index optical fibers based on CYTOP with a typical core diameter of 50 µm are commercially available.

The first inscription of an FBG in the graded-index multimode CYTOP fiber was reported in 2014 by Koerdt et al. [16,17]. By applying a CYTOP fiber with core diameter of 50 µm (GigaPOF 50 SR) and by removing the coating layer of the fiber by chemical etching with chloroform, an FBG with a grating period of around 1 µm was written in the polymer core using a krypton fluoride (KrF) excimer laser at 248 nm and the phase mask method. Several reflection peaks and transmission dips in the spectrum were detected using an optical spectrum analyzer (OSA) and a broadband light source as well as standard single-mode fibers for the light coupling. Following this, Lacraz et al. presented an FBG written in the same type of fiber but with a core diameter of 62.5 µm using a femtosecond laser [18]. With accurate control of the focused femtosecond laser beam spot and a motion stage, a grating with a period of 2.34 µm was directly inscribed in the CYTOP fiber core without removal of the coating. The reflection spectrum of the grating indicated several Bragg reflection peaks as well, which are due to the large refractive index change induced by non-linear multiphoton absorption and the formation of higher order Bragg reflections [19].

In this paper, we demonstrate for the first time the inscription of an FBG in a perfluorinated CYTOP based multimode graded-index fiber using a KrF excimer laser and a multimode interrogation setup. By applying multimode interrogation, the resulting FBG reflection spectrum contains only a single reflection peak with a FHWM bandwidth of about 9 nm. Moreover, investigation reveals that the spectral shape of the envelope of the single FBG reflection peak can be optimized by controlling the KrF excimer laser irradiation time. This feature combined with the multimode interrogation setup can be advantageous for new applications of the CYTOP-based POF in the area of low-cost multimode fiber-optic sensing, e.g., in structural health monitoring and observation of vital parameters in health care, or telecommunication. For this purpose, we have also investigated the strain, temperature, and humidity sensitivity of the polymer optical fiber exploiting FBGs as well as their failure strain.

## 2. Materials and Methods

### 2.1. Theoretical Background on Graded Index Multimode FBGs

Mizunami et al. [20] described theoretically and verified experimentally the spectral response of FBGs inscribed in graded index multimode fibers (GI-MMF). In GI-MMF, modes with the same propagation constant will form principal mode groups (PMGs) with discrete propagation constants [21]. When inscribing FBGs in GI-MMF, counter-propagating coupling of PMGs of the same order and of adjacent PMGs will occur and will cause discrete spectral peaks or dips in the FBG reflection or transmission spectrum, respectively. Depending on the difference of the propagation constants of the PMGs, the spectral response of the FBG spectrum can be controlled. For instance, when the difference of the propagation constants is sufficiently large, the resulting discrete reflection peaks or transmission dips are separated in the spectral domain. However, in the opposite case, i.e., for small propagation constant differences, the individual spectral reflection peaks or transmission dips overlap and thus the superposition will form a single spectral transmission dip or reflection peak. Furthermore, the amplitude of the discrete peaks and dips in the reflection and transmission spectra of the FBGs inscribed in the GI-MMF will depend on the length and refractive index modulation of the FBG as well as on the excitation of the individual fiber core modes. Theoretically, a steady-state and uniform excitation of the fiber core modes is usually assumed. However, in practice, the excitation of the fiber core modes might fluctuate due to, e.g., temporal variation of the coupling between the light source and GI-MMF, and consequently, the measured spectral shape of the FBG spectrum might change over time.

### 2.2. FBG Inscription and Optical Interrogator

For the inscription of the FBGs, a perfluorinated polymer optical fiber (GigaPOF 50SR, Chromis Fiberoptics, Warren, NJ, USA) was applied. The multimode graded-index CYTOP fiber has a core, cladding and coating diameter of 50 µm, approximately 80 µm and 490 µm, respectively. In the first step, the coating material of a fiber segment was removed using chloroform in order to inscribe FBGs into the fiber core. It was observed that during etching, the chloroform first softens the coating material and after about three minutes it separates the coating material from the GigaPOF 50SR. Thus, the total etching time to remove the coating was about three minutes, and the etching process can be visually controlled quite accurately without affecting the cladding of the fiber. Furthermore, since after removing of the coating material the remaining optical fiber still consists of a cladding and core material, the recoating of the fiber after the FBG inscription process is not required. However, due to the small cross-section, the handling of the etched polymer fiber was elaborate and care has to be taken to prevent damage.

**Figure 1:**
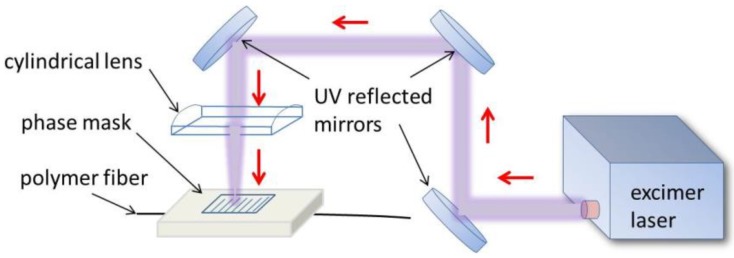
Experimental setup for the FBG inscription.

After completing the etching process, the end facets of the modified fiber segment were cleaved using a sharp razor blade. The treated fiber was then placed on a fiber-to-fiber launch stage and aligned accurately to a standard 50-µm multimode glass fiber (OM4). A small drop of index matching oil (*n* = 1.44) was used to reduce Fresnel reflection at the interface between the polymer and the glass fiber. Following this, a 4-mm-long FBG was written in the treated polymer optical fiber with the standard phase mask method utilizing a KrF excimer laser ATLEX-300-FBG (ATL Lasertechnik) and a phase mask (Ibsen Photonics A/S) with a grating period of 1070 nm and a length of 10 mm. A schematic of the optical setup used to inscribe the FBGs is shown in Figure 1. It consists of three mirrors (Thorlabs PF10-03-F01) to guide the laser light to the sample and one cylindrical lens (f = 100 mm, Thorlabs LJ4395) to focus the laser onto the sample and the phase mask. All three mirrors of the inscription setup were kept in fixed positions during the FBG inscription.

**Figure 2:**
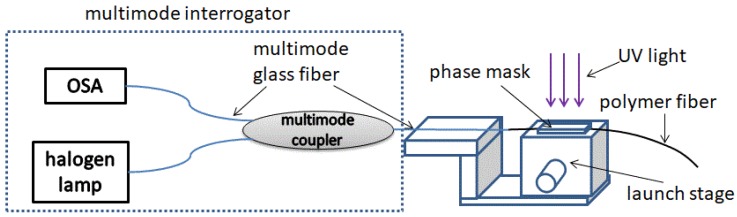
Setup to measure the reflection spectrum of the FBGs during inscription.

In light of the results reported in [16], the laser pulse repetition rate and pulse energy for the FBG inscription were chosen at 40 Hz or 20 Hz and 40 mJ/cm^2^, respectively. Higher pulse energies damaged the modified GigaPOF fiber. In order to characterize the evolution of the resulting reflection spectrum of the FBG during fabrication, a broadband light source (SLS201L/M, halogen lamp, Thorlabs, Newton, NJ, USA), a 50:50 fused fiber multimode coupler (all4fiber) as well as an OSA (AQ6317B, Ando, Kawasaki, Kanagawa, Japan) with a spectral resolution of 0.03 nm were applied. Furthermore, to determine the absolute FBG peak reflectivity, the measured reflected light power was calibrated to the light reflection that occurred at the glass/air interface of the OM4 multimode fiber pigtail. The applied optical multimode interrogator is illustrated in Figure 2. After the inscription process, the polymer fiber was kept at room temperature for 12 h to completely remove the thermal influence caused by the high laser pulse frequency.

### 2.3. Experimental Set-Up

#### 2.3.1. Evaluation of Strain Sensitivity

For the characterization of strain sensitivity, one end of the modified CYTOP fiber with FBG was mounted to a fiber-to-fiber launch stage and the other end to a linear stage with a positioning accuracy of 0.01 mm. In order to obtain a strong bond, both ends were glued to fiber holders using some drops of UV curable adhesive (NOA63, Norland, Cranbury, NJ, USA) and these were then screwed to the stages. The strain sensitivity was then evaluated by changing the length of the fiber under tension and measuring the resulting reflection spectrum of the FBG using the optical interrogator shown in Figure 2. Due to the relatively low reflected light intensities, a scanning time of 2 min was required per reflection spectrum scan. In a first attempt to characterize the sensor performance, a cross-correlation method was applied in Matlab (The MathWorks Inc., Natick, MA, USA) to calculate the Bragg wavelength shift. Since, according to Section 2.1 a spectrally broader and inhomogeneous FBG reflection peak is expected, a simple peak detection algorithm which is commonly applied for single-mode based FBGs is not sufficient to determine the wavelength shift of the Bragg reflection peak. For this purpose, the initial spectrum of the reflected FBG spectrum was recorded at the beginning of each measurement and then the cross-correlation between the actual and the recorded spectrum were calculated for each applied strain value of the measurement. Depending on the maximum of the resulting cross-correlation function, the central position of the Bragg wavelength was determined. Since the result of the cross-correlation function contained a single main peak, the relative shift of the reflected FBG spectrum was relatively easy to detect. Moreover, since polymers are viscoelastic materials and their creep and stress relaxation properties lead to a hysteresis [22], the sensor response to the applied strain was measured for both increasing and decreasing strain. Furthermore, the impact on the failure strain of the modified GigaPOF fiber with FBG was investigated as a function of the KrF excimer laser irradiation time.

#### 2.3.2. Evaluation of Temperature and Humidity Sensitivity

In order to characterize the temperature and humidity sensitivity, one end facet of the modified CYTOP fiber with FBG was glued to the end facet of a standard 50-µm multimode glass fiber pigtail (OM4) using UV curable adhesive (NOA63, Norland). To obtain a low-loss interconnection, the alignment of the CYTOP fiber relative to the 50-µm multimode glass fiber was controlled during the curing process by measuring the transmission power. Following this, the CYTOP fiber with the attached glass fiber pigtail was fixed in a perforated plastic cylinder, and the whole arrangement was then mounted in a humidity chamber (CTC256, Memmert, Aeussere Rittersbacher Strasse, Schwabach). The temperature and humidity sensitivity were evaluated using the same optical interrogator and the cross-correlation method explained in Section 2.3.1 for three subsequent temperature and humidity cycles.

## 3. Results and Discussion

### 3.1. Evolution of the FBG Spectrum during Fabrication

**Figure 3:**
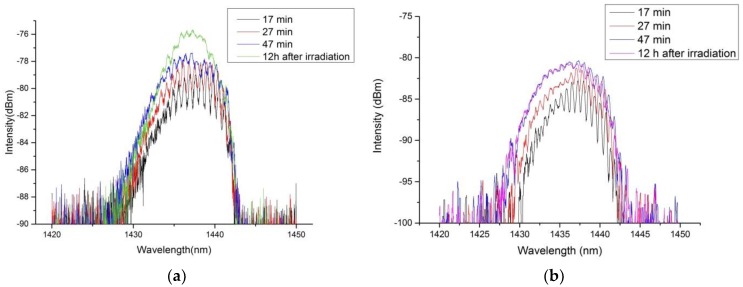
Evolution of the reflection spectrum during and after the irradiation process for two different samples, at 40 Hz (**a**) and 20 Hz (**b**) laser repetition rates.

The FBG reflection spectrum was monitored online using the optical interrogator shown in Figure 2 during the inscription method. The evolution of the Bragg reflection at different irradiation times for a KrF excimer laser repetition rate of 20 Hz and 40 Hz is exemplarily illustrated in Figure 3. From there it follows that the center wavelength of the Bragg reflection shifts towards the blue wavelength range during inscription. This blue shift can be explained by the negative thermo-optic coefficient of the fiber and the heat that is induced in the fiber due to the irradiation of the KrF excimer laser. Furthermore, according to Figure 3, a single Bragg reflection peak with an FWHM bandwidth of about 9 nm was observed after exposure of the fiber to KrF excimer laser light and storage at room temperature for 12 h. According to Section 2.1, the more pronounced sinusoidal modulation of the spectral intensity distribution of the Bragg reflection spectrum at the beginning of the laser irradiation can be explained by the different PMGs. In the case of Bragg gratings, ideally each PMG is represented by a single and narrow-bandwidth Bragg reflection peak. However, differences in the propagation constants of the modes within a PMG cause an increase of the spectral bandwidth of the Bragg reflection peak of each PMG. Thus, the intensity distributions of adjacent PMGs overlap and hence the superposition results in a single reflection peak with a sinusoidal modulation. With increasing KrF excimer laser irradiation, the bandwidth of the Bragg reflection peaks of the PMGs becomes even broader due to the increase in the modulation depth of the refractive index, and consequently, the amplitude of the sinusoidal modulation decreases. Moreover, the resulting single Bragg reflection peaks illustrated in Figure 3 show a slight asymmetry, which can be explained by the different excited intensities of the PMGs. The final reflectivity of the FBG reflection spectra shown in Figure 3a,b are 50% (at 40 Hz) and 16% (at 20 Hz), respectively. The difference in the FBG reflection peaks is due to the different KrF excimer laser irradiation doses and thus the difference in the accumulated refractive index modulation of the fiber core. Furthermore, compared to the bandwidth of FBGs inscribed in Germanium (Ge)-doped fused silica GI-MMFs (16 nm [20]), the bandwidth of the FBG reflection spectrum here is relatively small (in the order of 9 nm). The smaller bandwidth evolves as the differences of the propagation constants of adjacent PMGs are smaller.

With regard to applications in fiber optic sensing or telecommunication, the resulting single Bragg reflection peak can be advantageous. For instance, due to the large cross-section of the multimode POF, the coupling of light sources to the optical fiber is less complicated and, thus, low-cost and broadband LED sources can be applied as light sources. In addition, the single multimode Bragg reflection peak can also be advantageous in terms of multimode fiber optic communication application and wavelength division multiplexing (WDM).

### 3.2. Sensitivity to Applied Strain

When strain is applied to the fibers under test, an increase of the grating period and a change in the effective refractive index due to the strain-optic effect can be observed. Both effects lead to a red shift of the Bragg wavelength. In Table 1 the strain sensitivity and the impact on the failure strain of the modified GigaPOF 50SR fibers with FBGs are summarized for different KrF excimer laser irradiation times and a constant pulse energy of 40 mJ/cm^2^. We find that with increasing UV-light irradiation time, the failure strain decreases whereas the sensitivity to the applied strain is almost constant.

**Figure 4:**
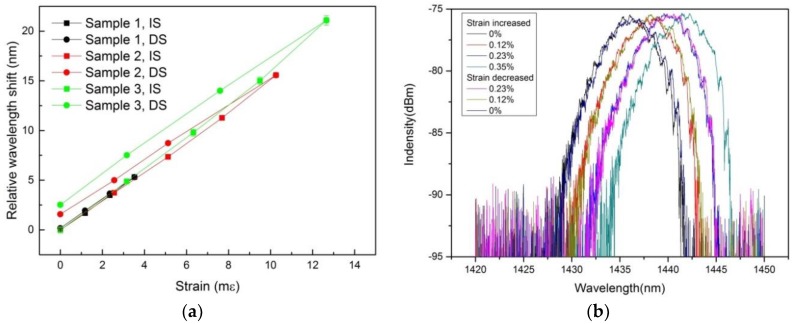
Response of the Bragg wavelength to increasing and decreasing strain, IS and DS, respectively, for three different samples (**a**) and illustration of the shift of the Bragg wavelength due to applied strain (**b**).

In Figure 4a the response of the different samples to increasing and decreasing applied strain, IS and DS, respectively, as well as the evolution of the sensor hysteresis depending on applied strain are illustrated. Here, the term failure strain is used instead of tensile strength since at the time of the measurements the experimental setup was not able to determine the Young modulus. Based on the results in Table 1 and the observation when conducting the experiments, no hysteresis effect was observed for applied strain values equal or less than 8 mε, which is consistent with results reported in [23]. In addition, since polymers are compliant materials with a low elastic module, the hysteresis can be reduced when the sensor is attached directly to a structure with elastic material properties [24]. Figure 4b exemplarily depicts the change of the multimode Bragg reflection peak of Sample 1 for different applied strain values. For this purpose, the applied strain was slowly increased to a value of 3.5 mε and then decreased slowly back again to the original value. The obtained strain sensitivity in the order of 1.5 nm/mε is in good agreement with the strain sensitivity reported in [18] and represents a roughly 20% sensitivity increase compared to PMMA-based POFs, which display a typical strain sensitivity of 12–13 nm/% in the 1435-nm range [25].

**Figure 5:**
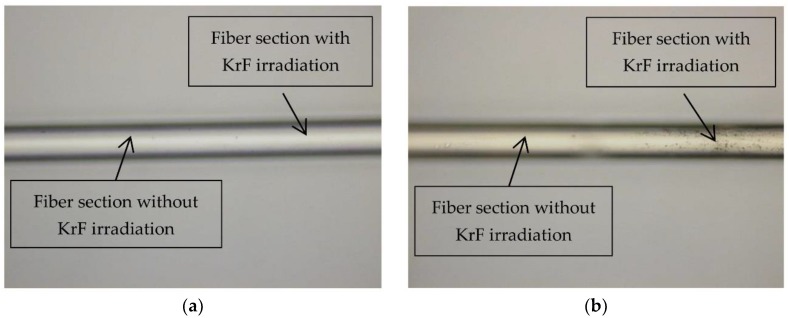
Surfaces of modified CYTOP fibers with FBGs after exposure to KrF excimer laser irradiation of 4 mJ/cm^2^ and 20 Hz for 10 min (**a**) and for 30 min (**b**), respectively.

The surfaces of the modified CYTOP fibers after FBG inscription were investigated in order to elucidate the reason for the reduction of the failure strain. In Figure 5, images of the surfaces of different modified CYTOP fibers with FBGs are illustrated. The images were taken with a Keyence VK-X200 microscope and a magnification of 20. For the illustrated fibers, different inscription parameters were applied. Under a short exposure time (10 min) and low pulse repetition rate of 20 Hz, no modification or ablation at the fiber surface is observed (Figure 5a). However, as shown in Figure 5b, when the exposure time increases (30 min) the fiber surfaces were ablated, which is the reason for the reduction of the failure strains of the fibers under investigation.

### 3.3. Sensitivity to Applied Temperature

**Figure 6:**
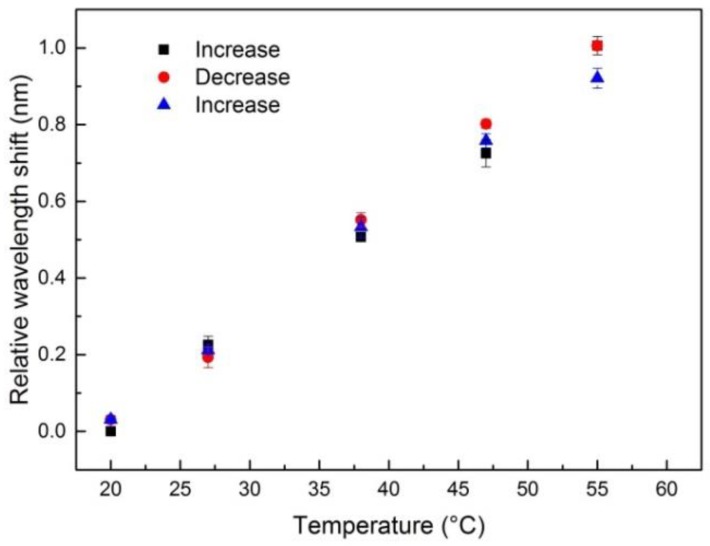
Temperature response of the multimode POF FBG sensor.

The characterization of the temperature sensitivity was performed at a constant relative humidity (RH) of 50%. The temperature sensitivity of the modified CYTOP fiber with FBG was measured in the temperature range from 20 °C to 55 °C in three subsequent cycles. During the measurement , a decrease of the amplitude of the Bragg reflection peak was observed. It is assumed that this decrease is due to the thermal expansion of the interconnection between the multimode glass fiber and the multimode POF. In Figure 6 the shift of the Bragg wavelength is illustrated for different applied temperatures. The CYTOP fiber with FBG exhibits a temperature sensitivity of 27.5 ± 2.4 pm/K in the temperature range from 20 °C to 55 °C, which is consistent with the temperature sensitivity reported in [17]. Furthermore, no hysteresis was observed for the three subsequent temperature cycles. According to [26], the Bragg wavelength has a linear dependence on both the temperature expansion coefficient and thermo-optic coefficient. Both coefficients can be assumed constant over a sufficiently small temperature range (about 15 °C for PMMA) [27]. In addition, polymers usually have a positive expansion coefficient and a negative thermo-optic coefficient. Thus, the Bragg wavelength shifts towards the blue or red spectral range depending on which coefficient is more dominant. Bulk CYTOP has a thermo-optic coefficient and an expansion coefficient of −5.0 × 10^−5^/K and 7.4 × 10^−5^/K, respectively [18]. Therefore, for the CYTOP fiber, a red shift of the Bragg wavelength is expected with increasing temperature. For instance, for a Bragg wavelength at approximately 1435 nm the temperature sensitivity would be approximately 34 pm/K. This agrees well with the measured temperature sensitivity of 27.5 ± 2.4 pm/K in the temperature range from 20 °C to 55 °C.

### 3.4. Sensitivity to Applied Humidity

**Figure 7:**
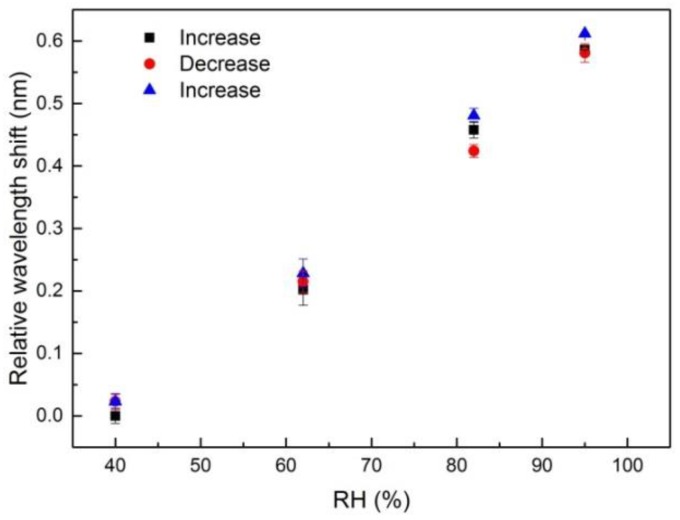
Humidity response of the multimode POF FBG sensor.

In general, CYTOP has a weak affinity to water and is, thus, usually considered as a potentially humidity insensitive material. In this work, we investigated the sensitivity of the modified CYTOP fiber with FBG to applied relative humidity (RH) in the humidity range from 40% RH to 95% RH at a constant temperature of 36 °C. As shown in Figure 7, the Bragg wavelength of the fiber under test shows a linear dependence on applied humidity with a sensitivity of 10.3 ± 1.8 pm/RH. Moreover, no hysteresis was observed for the three subsequent humidity cycles. Zhang et al. demonstrated that the response time to different humidity levels of FBGs inscribed in POFs depends on the fiber diameter and molecular structure and can be as large as a few tens of minutes [28]. For our multimode fiber, the response time to different humidity levels was less than two minutes, which renders the material attractive for humidity sensing applications.

## 4. Conclusions

In this work, we inscribed FBGs into perfluorinated multimode CYTOP fibers (GigaPOF 50SR) by using the well-known phase mask method, a KrF excimer laser at 248 nm and inscription times of several ten minutes. By applying a multimode optical interrogator, a single Bragg reflection peak with a FWHM bandwidth of about 9 nm was obtained, and the shape of the FBG reflection spectrum can be controlled by the irradiation time of the KrF excimer laser. The sensitivity of the modified GigaPOF 50SR fiber with FBG to applied strain, temperature and humidity was carefully investigated. The characterization verifies a linear response to all three quantities with sensitivities in the order of 1.5 nm/mε, 27.5 ± 2.4 pm/K and 10.3 ± 1.8 pm/RH to applied strain, temperature and humidity, respectively. For strain values above 8 mε, a hysteresis effect was observed. Moreover, the failure strain of the modified fiber depends on the duration and pulse energy of the KrF excimer laser irradiation. It was observed that long inscription times and high pulse energies reduce the failure strains of the fibers under test. Consequently, in terms of strain sensing, a trade-off between spectral behavior and strain sensing range has to be made. However, when only the measurement of temperature and/or humidity is required, the control of the irradiation time of the KrF excimer laser is less critical. Since the CYTOP GigaPOF 50SR fiber has low light attenuation in the near infrared wavelength range, the reported sensor can be applied in current data transmission systems and sensing networks. The large cross-section of the multimode fiber core also simplifies the light coupling since no sophisticated alignment and optical free-space coupling systems are required. Hence, in order to interrogate the modified GigaPOF fiber with FBG, relatively low-cost and broadband LEDs as well as simple detectors such as grating-based spectrometers or even photodiodes, can be applied. This feature will allow new applications in fiber optic sensing, e.g., in structural health monitoring and patient supervision in medicine. Moreover, since the modified CYTOP fiber with FBG is inherently sensitive to strain, temperature and humidity, common techniques such as reference sensors and/or an appropriate sensor packaging can be applied in order to compensate for cross-sensitivities when only one parameter has to be measured. In terms of fiber optic communication, the single FBG reflection peak can also pave the way towards novel wavelength division multiplexing (WDM) applications in multimode fiber optic telecommunication systems. In future work, Young’s modulus of the treated/untreated GigaPOF 50 SR will be investigated. Also, the signal processing of the broad multimode FBG reflection peak and its longterm stability, i.e., sensitivity to a non-steady-state modal excitation and longterm degeneration of the polymer fiber core refractive index modulation, the sensitivity to applied humidity at different temperatures as well as the multiplexing capability and the integration into composite structures for structural health monitoring, will be studied.

## Figures and Tables

**Table 1:** Obtained strain sensitivities and failure strains for different KrF excimer laser parameters.

Sample	Irradiation Time (min)	Repetition Rate (Hz)	Sensitivity (nm/mε)	Failure Strain (mε)	Sample Length (mm)
1	47	40	1.506 ± 0.021	7	78
2	30	20	1.509 ± 0.071	18	79
3	10	20	1.577 ± 0.097	25	79
